# Neuroendocrine Neoplasms of the Esophagus and Esophagogastric Junction in Germany, 2009–2023

**DOI:** 10.3390/curroncol33020101

**Published:** 2026-02-04

**Authors:** Andreas Stang, Ina Wellmann, Bernd Holleczek, Alice Nennecke, Guido Schumacher, Hiltraud Kajüter

**Affiliations:** 1 Institute of Medical Informatics, Biometry, and Epidemiology, University Hospital Essen, Hufelandstr. 55, 45147 Essen, Germany; 2 Cancer Registry of North Rhine-Westphalia, Gesundheitscampus 10, 44801 Bochum, Germany; ina.wellmann@krebsregister.nrw.de (I.W.); hiltraud.kajueter@krebsregister.nrw.de (H.K.); 3 Saarland Cancer Registry, Neugeländstr. 9, 66117 Saarbrücken, Germany; b.holleczek@krebsregister.saarland.de; 4 Hamburg Cancer Registry, Süderstr. 30, 20097 Hamburg, Germany; alice.nennecke@bgv.hamburg.de; 5 Department of General Surgery, Hospital of Brixen (SABES), Dantestr. 51, 39042 Brixen, Italy; guido.schumacher@sabes.it

**Keywords:** neuroendocrine tumors, carcinoma, neuroendocrine, neoplasm grading, incidence, survival, cancer registries, Germany

## Abstract

**Background**: The influence of the grading of esophageal neuroendocrine neoplasms (NENs) on prognosis has so far only been investigated in small groups. The aim was to determine the incidence and relative survival of NENs and its subtypes of the esophagus. **Methods**: We included incident cancers of the esophagus and esophagogastric junction (EGJ) for the years 2009–2023, registered by German cancer registries. NENs were subdivided into neuroendocrine tumors (NETs), neuroendocrine carcinomas (NECs), and mixed forms (MiNENs). To compare the epidemiology of NENs with that of adenocarcinoma and squamous cell carcinoma, we also evaluated these entities in a statistically identical manner. We estimated age-standardized incidence rates (cases per million person-years, old European standard) and 5-year relative survival (period analysis). **Results**: Of the 2025 NENs of the esophagus and EGJ, 69.9% were NECs, 19.5% were NETs, and 10.7% were MiNENs. 45.0% of NENs occurred at the EGJ. The mean age at diagnosis of esophageal NEN was 67 years. The age-standardized incidence of NEN and the subgroups increased over time, with NEN incidences of 1.1 and 0.4 in 2019 and 2023. Relative survival based on 1320 NENs was 22.8%, with NETs having a markedly better prognosis (59.8%) than NECs (12.5%) and MiNENs falling in between (25.3%). Patients with NET-G1 tumors had a relative survival of 83.8%, while patients with NET-G2 and NET-G3 tumors had relative survival of 50.0% and 9.0%, respectively. **Conclusions**: Even though accurate diagnosis and coding of NENs is difficult, grading of esophageal NETs has a strong influence on prognosis. Esophageal and esophagogastric NECs have a poorer 5-year relative survival than adenocarcinomas and squamous cell carcinomas.

## Simple Summary

Until recently, the influence of the grading of neuroendocrine tumors (G1–G3) of the esophagus on prognosis could not be assessed due to limited data. We used all cancer registry data from Germany (population of 83 million people) in order to assemble the largest possible cohort of patients with these tumors and included 2025 newly diagnosed patients with esophageal or esophagogastric neuroendocrine neoplasms (394 neuroendocrine tumors (NET), 1415 neuroendocrine carcinomas, and 216 mixed neuroendocrine neoplasias in Germany 2009–2023. In the survival analyses of 1320 neuroendocrine neoplasms, we found that the grading of NETs has a strong influence on prognosis. Patients with NET-G1 tumors had a relative 5-year survival of 83.8%, while patients with NET-G2 and NET-G3 tumors had relative survival of 50.0% and 9.0%, respectively. Relative 5-year survival was 12.5% and 25.3% for neuroendocrine carcinomas and mixed neuroendocrine neoplasms, respectively.

## 1. Introduction

According to the WHO classification of tumors, neuroendocrine neoplasms (NENs) of the esophagus are esophageal “epithelial neoplasms with neuroendocrine differentiation, including well-differentiated neuroendocrine tumours (NETs), poorly differentiated neuroendocrine carcinomas (NECs), and mixed neuroendocrine–non-neuroendocrine neoplasms (MiNENs)—an umbrella category including mixed adenoneuroendocrine carcinoma (MANEC)” [1].

Due to the rarity of these tumors (0.04–1% of all gastroenteropancreatic NENs), only a few epidemiological and etiological studies have been conducted on these tumors to date. As recently as 2019, the WHO classification of tumors stated that fewer than 50 cases of esophageal NETs had been reported in the literature. Furthermore, the WHO classification stated that the influence of the grading of NETs of the esophagus could not be assessed due to limited data. NECs are by far the most common NENs in the esophagus. There is no specific TNM staging for esophageal NENs, and esophageal NETs, NECs, and MiNENs are staged in the same way as esophageal carcinomas. Esophageal NENs are graded according to the same criteria as gastroenteropancreatic NENs using mitotic count and the Ki-67 index as criteria [1,2].

The aim of this study was to use the Germany-wide dataset from all cancer registries to determine the incidence and survival probabilities of NENs and its subtypes of the esophagus and esophagogastric junction. Although the focus of this study is on NENs of the esophagus and esophagogastric junction, we also present statistically identical analyses for adenocarcinomas and squamous cell carcinomas of the esophagus and esophagogastric junction (Supplementary Materials only) to facilitate epidemiological comparison with these histologies.

## 2. Materials and Methods

Due to regulation, every Federal State in Germany has an operating cancer registry that records the incidence of cancer across the state. Physicians are required by law to report to their cancer registry if they diagnose or treat cancer. Since multiple physicians are often involved in the treatment, there are at least three types of reporting sources (diagnosis, pathology, and therapy) per cancer, with an average of five to ten reports per cancer. A unified national oncology dataset that is legally binding serves as the foundation for the cancer documentation for the cancer registry reports. Additionally, death data from civil registries is routinely sent to the cancer registries. Data from the state cancer registries are gathered together in the Center for Cancer Registry Data at the Robert Koch Institute. Additional information about Germany’s cancer registration has been provided elsewhere [3]. We used the combined national data file from all population-based cancer registries in Germany from 2009 to 2023, which included 83 million at-risk individuals as of 2023. The Robert Koch Institute’s Center of Cancer Registry Data supplied this file. All incident instances of primary malignant tumors of the esophagus (ICD-10 [4], C15, *International Statistical Classification of Diseases and Related Health Problems, 10th edition*) and esophagogastric junction (ICD-10: C16.0), abbreviated as EGJ, were retrieved.

Cancer morphologies, coded by the *International Classification of Diseases for Oncology (ICD-O), 3. Edition* [5], were grouped into adenocarcinoma (M8140/3-M8149/3, M8160/3-M8162/3, M8190/3-M8221/3, M8260/3-M8337/3, M8350/3-M8551/3, M8570/3-M8576/3, M8940/3-M8941/3), squamous cell carcinoma (M8051/3-M8084/3, M8085/3-M8086/3, M8120/3-M8131/3), neuroendocrine tumors (NENs) (M8240/3-M8242/3, M8249/3), neuroendocrine carcinomas (NECs) (M8013/3, M8041/3, M8246/3), mixed neuroendocrine–non-neuroendocrine neoplasms (MiNENs), previously termed as mixed adenoneuroendocrine carcinoma (MANEC) (M8154/3, M8244/3), and other or unspecified cancers. The 1497 tumors coded as NECs consisted of 232 large-cell NECs (M8013/3), 569 small-cell NECs (M8041/3), 678 NECs not otherwise specified (M8246/3). Overall, 64 (4.3%) were reported with a grade of I-II (4 large-cell NECs, 18 small-cell NECs, 42 NECs not otherwise specified). These were reclassified as NETs.

To compare the epidemiology of NENs with that of adenocarcinomas (ACs) (M8140-M8149, M8160-M8162, M8190-M8221, M8260-M8337, M8350-M8551, M8570-M8576, M8940-M8941) and squamous cell carcinomas (SCCs) (M8051-M8086, M8120-M8131) of the esophagus and EGJ, we also extracted these histological entities of the esophagus and EGJ from the same dataset and evaluated them in a statistically identical manner to the NENs. Based on the reported TNM or UICC stage, we derived a coarser staging classification based on the American Joint Committee on Cancer’s (AJCC) system that allowed us to differentiate between localized cancers, cancers with regional lymph node metastases, and cancers with distant metastases, respectively.

### Statistical Methods

By using the annual population figures for age and sex, we calculated the annual and overall age-standardized incidence rates (ASRs) for the overall registration period in Germany by sex by using the old European standard population for age standardization [6]. To estimate the incidence trend of NENs for the years 2009–2023, we stratified the age-standardized incidence rates neither by sex nor by anatomical location (esophagus, EGJ) in order to achieve greater precision in the trend estimation. We fitted a segmented linear model to the ASR data with a joinpoint regression program [7]. The software tests for changes in slope, with a minimum of 0 and a maximum of 5 joinpoints. The minimal distance from the first observation of the time series to the first joinpoint (resp. from the last joinpoint to the last observation) was 2 data points [8].

Relative 5-year survival (RS) was calculated as the ratio of the patients’ observed survival to the expected survival of a group of German citizens with an average risk of death who were matched for sex, age, and calendar time. In other words, an RS estimate of 100% signifies that the patients’ observed mortality is the same as their expected mortality in the general population [9]. RS was computed using an SAS macro (SAS 9.4 for Windows, SAS Institute, Cary, NC, USA) in accordance with the methodology of Brenner et al. [10]. The follow-up period ended on 31 December 2023. The most recent survival estimates were obtained using period analysis methods [11,12,13]. The period analysis method made use of the survival observations that were available between 2009 and 2023.

We also report the absolute 5-year survival probabilities (period approach) for the interested reader in the Supplementary Materials. In contrast to the incidence analysis, we excluded cancer registries that had problems with vital status monitoring from the survival analyses. All survival time analyses are based on data from the cancer registries of Schleswig-Holstein, Hamburg, Lower Saxony, Bremen, North Rhine-Westphalia, Rhineland-Palatinate, Baden-Württemberg, Saarland, and Saxony. Death-certificate-only (DCO) cases were excluded from survival analyses.

To estimate the AJCC, sex, calendar period, and age-adjusted effect of NET grading on absolute survival, we performed multiple imputations in addition to a complete case analysis. We used the missing at random assumption to replace missing values. Statistical details of the multiple imputation can be found in the Supplementary Materials. We calculated and report standard errors (SEs) to assess the precision of our estimates because our goal is estimation and not significance testing. We wish to avoid publication bias by preferential reporting of statistically significant results. Instead, we judge the value of our estimates by their precision and validity [14,15].

## 3. Results

Between 2009 and 2023, 2025 NENs of the esophagus and EGJ were registered. Overall, the cancer registries had information that 95.2% of all NENs had been histologically verified and 15 cases (0.7%) were death-certificate-only cases. NENs of the esophagus and EGJ were most frequently NECs (69.9%), followed by NETs (19.5%) and MiNENs (10.7%). If unspecified NECs (45%) are excluded, the proportion of small-cell NECs in the esophagus is markedly greater than that of large-cell NECs. This difference is smaller in NECs of the EGJ. Although the EGJ is anatomically a much smaller area than the esophagus, 45.0% (911 out of 2025) of all NENs occurred at the EGJ. Among all NENs, the percentage of NECs was higher in the esophagus than in the EGJ (81.8% versus 55.3%, difference 26.5 percentage points, 95%CI 22.5; 30.4). The relative frequency of MiNEN (8.4%) was lower in the esophagus than in the EGJ (13.4%) (difference −5.0 percentage points, 95%CI −7.8; −2.2). NETs occurred with a lower percentage in the esophagus (9.8%) than in the EGJ (31.3%) (difference −21.5 percentage points, 95%CI −25.5; −18.5). NETs of the EGJ were more frequently of low grade (G1, 55.4%) than NETs of the esophagus (G1, 19.3%) (difference 36.1 percentage points, 95%CI 26.2; 45.1). The percentage of NETs among all NENs in the esophagus and EGJ was higher in women than in men (31.0% versus 14.9%, difference 16.1, 95%CI 11.9; 20.4), while the proportion of MiNENs was lower in women than in men (6.5% versus 12.3%, difference −5.8 percentage points, 95%CI −8.4; −3.1). Within the group of NETs of the esophagus and EGJ, women had a greater proportion of G1 tumors than men (59.3% versus 34.1%, difference 25.2 percentage points, 95%CI 15.5; 34.6) (Table 1). A complete case analysis of the subgroup of NETs for which both grading and AJCC stage were available (88 Patients) showed that G1 was present in 55.9% of localized NETs, while this proportion was only 10.0% and 12.8% for regionally metastatic NETs and distantly metastatic NETs, respectively. After multiple imputations, these proportions for NET-G1 were 74.8%, 30.5%, and 25.2% respectively.

The detailed study of the anatomical distribution of NENs and NEN subgroups within the esophagus showed that NENs as a whole and MiNENs in particular occur preferentially in the lower part of the esophagus. Only adenocarcinomas showed an even stronger preference for the lower part of the esophagus. Squamous cell carcinomas, on the other hand, showed a more even distribution within the esophagus (Table 2).

The mean age at diagnosis of male and female patients with esophageal NENs (approximately 67.0–67.3 years) differed little from the mean age at diagnosis of patients with AC (67.3–68.2 years) and SCC (67.5–68.3 years). At most, women with EGJ NENs (68.7 years) had a noticeably lower age at diagnosis than those with SCC (72.2 years) and AC (71.6 years) (Supplementary Figure S1).

**Figure 1:**
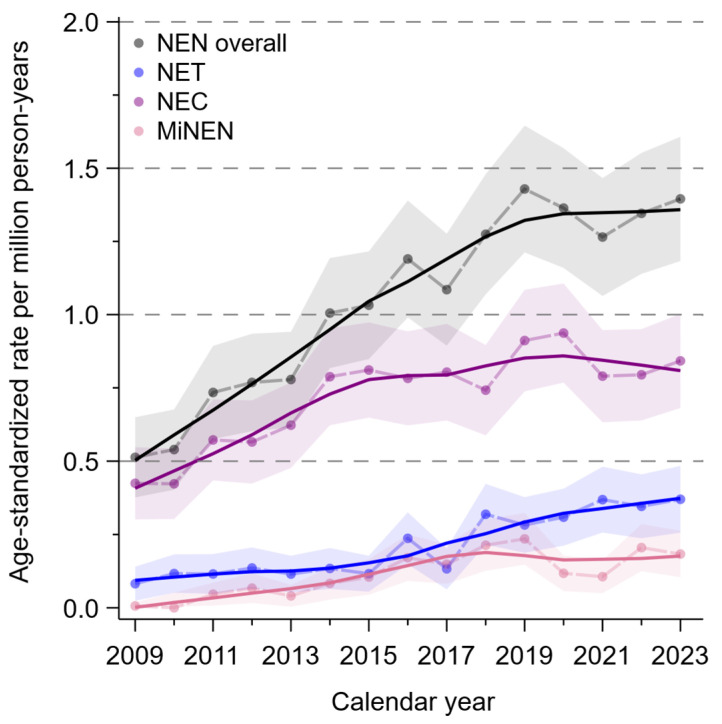
Incidence time trend of neuroendocrine neoplasms of the esophagus and esophagogastric junction in Germany, 2009–2023. Analyses include all German cancer registries; smooth solid trend lines by LOESS, color bands display 95% confidence intervals of the annual rates; dashed lines: unsmoothed trends; NEN: neuroendocrine neoplasm, NET: neuroendocrine tumor, NEC: neuroendocrine carcinoma, MiNEN: mixed neuroendocrine-non-neuroendocrine neoplasm, previously termed as mixed adenoneuroendocrine carcinoma (MANEC); the joinpoint regression analysis revealed a joinpoint for NEN overall and for NECs in 2016 (95%CI 2012–2020) and 2015 (95%CI 2013–2018) respectively.

Between 2009 and 2023, there was an increase in the age-standardized incidence of NENs overall and also in the subgroups (NET, NEC, MiNEN) nationwide. The relative increase in rates slowed down from around 2019 onwards. The formal joinpoint regression analysis revealed a joinpoint for NENs overall and for NECs in 2016 (95%CI 2012–2020) and 2015 (95%CI 2013–2018) respectively (Figure 1).

The latest crude and age-standardized incidence rates for neuroendocrine neoplasms, squamous cell carcinoma, and adenocarcinoma of the esophagus and EGJ (2019–2023) are presented in Table 3. With rates of 1.1 and 0.4 per million person-years in men and women for esophageal NENs compared to 45.2 and 7.4 per million person-years for adenocarcinomas, NENs in the esophagus are extremely rare. The age-standardized incidence rates of NETs at the EGJ are of the same order of magnitude as in the esophagus. In general, men had higher age-standardized incidence rates of primary malignant neoplasms of the esophagus and EGJ than women. In particular, men had a much higher incidence of adenocarcinomas of the esophagus and EGJ (esophagus: men 45.2 per million person-years, women 7.4 per million person-years, EGJ: men 50.5 per million person-years, women 11.3 per million person-years). The age-standardized incidence rates for NENs of the esophagus and EGJ were also higher in men than in women. The sex ratios (men/women) of the crude NEN incidence rates for the esophagus and EGJ were 2.4 and 2.3, respectively. The corresponding ratios of age-standardized rates were both 2.3 for the esophagus and EGJ.

**Figure 2:**
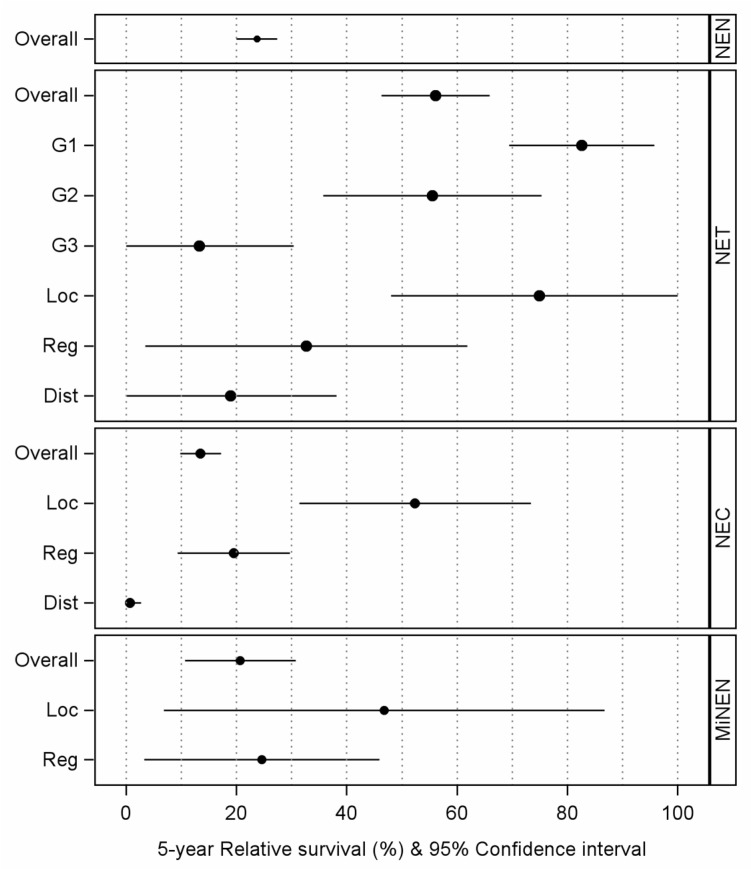
Relative 5-year survival of patients with neuroendocrine neoplasms of the esophagus and esophagogastric junction in Germany, 2009–2023 (period approach). Legend: Overall = includes all patients of the entity, Loc = localized cancers, Reg = cancers with regional lymph node metastases, Dist = cancers with distant metastases; G1–G3 indicates grading of NETs; patients with missing data are not displayed but are reported in the Supplementary Materials; only survival data from Schleswig-Holstein, Hamburg, Lower Saxony, Bremen, North Rhine-Westphalia, Rhineland-Palatinate, Baden-Wurttemberg, Saarland, and Saxony is included.

For the entire group of NENs, the 5-year RS was 22.8%, with NETs having a markedly better prognosis (59.8%) than NECs (12.5%) and MiNENs falling in between (25.3%). The grading of NETs was strongly associated with the 5-year RS. Patients with NET-G1 tumors had a 5-year RS of 83.8%, while patients with NET-G2 and NET-G3 tumors had 5-year RS of 50.0% and 9.0%, respectively. This puts the 5-year RS of NET-G3 tumors in the same range as NECs. Even though the estimates for the association of 5-year RS with staging are based on imprecise estimates (wide confidence intervals), staging has a clear effect on the 5-year RS of NETs and NECs (Figure 2). The exact numerical results for the 5-year absolute and relative survival are presented in Supplementary Table S1. The complete case analysis and multiple imputations on the relationship between NET grading and absolute survival show that even after adjusting for AJCC, sex, calendar period, and age, there is a clear association between grading and absolute survival (Supplementary Text and Supplementary Tables S2–S4).

Results of comparable analyses of adenocarcinoma and squamous cell carcinoma of the esophagus and EGJ are presented in Supplementary Tables S5 and S6.

## 4. Discussion

Similar to others [16,17], we found that esophageal NETs, NECs, and especially MiNENs occur preferentially in the lower third of the esophagus, which may be due to the presence of endocrine cells in the submucosal glands of the lower esophagus or in Barrett’s mucosa [1]. Similar to the US SEER data (1975–2016) (small cell NEC 60.4%, NEC not otherwise specified 33.1%) [17], the most common histological subtype of NECs in the esophagus in Germany is small-cell NEC (46.7%), with NECs not otherwise specified accounting for 40.6%. A more recent analysis of US SEER data (1975–2021) showed that, after excluding MiNENs, small-cell NECs accounted for 56.5% and NECs not otherwise specified accounted for 36.4% [18].

In the WHO classification, Scoazec and Rindi stated that the mean patient age at diagnosis of esophageal NENs is 56 years [19]. However, our population-based German-wide analyses showed a considerably higher average age of diagnosis of 67 years. The average age of diagnosis for esophageal NECs observed in Germany is consistent with the results of the US SEER Program evaluation for the years 1975–2016, in which the average age of onset for 686 patients with esophageal NECs was 67 years [20]. The SEER analyses for the years 1975–2021 also showed a median age at diagnosis of 65 years [18]. Our estimated age-standardized incidence rate for esophageal NEN (2009–2023) was 1.1 and 0.4 per million person-years for men and women (old European standard population) respectively and is thus comparable in magnitude to the SEER program (2000–2016) with 0.44 per million (men and women combined, US 2000 population standard) [20]. While the WHO classification of tumors [1] refers to an incidence sex ratio (men/women) of esophageal NENs of 6:1, we found ratios of only 2.4:1 (crude incidence rates) and 2.8:1 (age-standardized rates), respectively.

It is unclear whether the increase in the age-standardized incidence of NENs and the imprecisely estimated joinpoints (year 2016 for NEN overall and 2015 for NECs) observed in Germany between 2009 and 2023 reflects a true increase or is an artifact due to, among other things, improved reporting by physicians, more specific information in pathology reports, and more specific coding of histopathological findings. A data analysis of the US SEER program for esophageal NECs revealed an annual increase of +1.272% between 1975 and 2016 [20]. In a further analysis, which did not focus specifically on NENs of the esophagus but on all small cell carcinomas of the esophagus, Zhu et al. initially found a decline in age-standardized incidence between 2005 and 2012, followed by an increase again until 2016 [21].

In Germany, as in other countries [22], esophageal and esophagogastric NECs were also found to have a poorer 5-year relative survival than adenocarcinomas and squamous cell carcinomas. Our work shows that the WHO-proposed G1–G3 grading of well-differentiated NETs of the esophagus and EGJ has a strong prognostic effect. In our data, grading was associated with AJCC staging. Various statistical analyses (complete case and multiple imputations with multiple adjustments) showed that after adjusting for AJCC stage and other potential confounders, a clear association between grading and survival remained despite the high proportion of missing information on AJCC stage.

### Limitations

Our study suffers from several limitations. First, the inherent problem of accurately diagnosing and coding of NENs and their subtypes, which is related to changing classifications, will have led to an unknown proportion of misclassifications of NEN subgroups and may have influenced the incidence time trend observed in Germany. For example, a new category was introduced in 2019—well-differentiated grade 3 NETs—which have since been officially separated from NECs [23,24]. MiNENs can be mistakenly classified as adenocarcinoma or squamous cell carcinoma with “neuroendocrine features” [25]. Second, in 10.7% and 45.0% of all NETs and NECs, respectively, grading or subtyping of NECs into small-cell and large-cell NECs was missing. The lack of NEC subtyping is also a known problem in US data for the years 2006–2014, where subtyping was missing in 42.8% of cases [26]. Third, as some German cancer registries temporarily experienced difficulties in reconciling vital status data, we had to exclude some cancer registries from the survival analyses, which led to less accurate survival estimates, especially for NENs. Fourth, the interpretation of incidence trends for tumors of the EGJ depends, among other things, on correct anatomical-topographical classification. A validation study based on data from the Swedish Cancer Registry for the years 1989–1994 showed that the accuracy of the registration of these tumors is so low that the true incidence of these tumors would be 15% lower to 45% higher than reported by the Cancer Registry [27].

## 5. Conclusions

We analyzed one of the largest population-representative cohorts of patients with NETs of the esophagus and EGJ and were able to quantify precise differences in 5-year relative survival. Survival was considerably better for NETs than for NECs and MiNENs. Even after adjusting for tumor stage, grading of NETs has a strong effect on survival, which supports the validity of the grading system, although there are still uncertainties in the diagnosis and coding of NENs.

## Figures and Tables

**Table 1:** Neuroendocrine neoplasms of the esophagus and esophagogastric junction in Germany, 2009–2023.

	Total		Men		Women	
Entity	N	%	N	%	N	%
**Esophageal & EGJ**						
NEN	2025		1454		571	
NET	394	19.5	217	14.9	177	31.0
NET, G1 *	179	45.4	74	34.1	105	59.3
NET, G2 *	108	27.4	71	32.7	37	20.9
NET, G3 *	65	16.5	48	22.1	17	9.6
NET, G unknown *	42	10.7	24	11.1	18	10.2
NEC	1415	69.9	1058	72.8	357	62.5
LCNEC *	228	16.1	169	16.0	59	16.5
SCNEC *	551	38.9	396	37.4	155	43.4
NEC, nos. *	636	45.0	493	46.6	143	40.1
MiNEN	216	10.7	179	12.3	37	6.5
**Esophagus**						
NEN	1114		810		304	
NET	109	9.8	72	8.9	37	12.2
NET, G1 *	21	19.3	9	12.5	12	32.4
NET, G2 *	39	35.8	30	41.7	9	24.3
NET, G3 *	32	29.4	24	33.3	8	21.6
NET, G unknown *	17	15.6	9	12.5	8	21.6
NEC	911	81.8	658	81.2	253	83.2
LCNEC *	116	12.7	85	12.9	31	12.3
SCNEC *	425	46.7	295	44.8	130	51.4
NEC, nos. *	370	40.6	278	42.3	92	36.4
MiNEN	94	8.4	80	9.9	14	4.6
**Esophagogastric junction**						
NEN	911		644		267	
NET	285	31.3	145	22.5	140	52.4
NET, G1 *	158	55.4	65	44.8	93	66.4
NET, G2 *	69	24.2	41	28.3	28	20.0
NET, G3 *	33	11.6	24	16.6	9	6.4
NET, G unknown *	25	8.8	15	10.3	10	7.1
NEC	504	55.3	400	62.1	104	39.0
LCNEC *	112	22.2	84	21.0	28	26.9
SCNEC *	126	25.0	101	25.3	25	24.0
NEC, nos. *	266	52.8	215	53.8	51	49.0
MiNEN	122	13.4	99	15.4	23	8.6

Legend: analyses include all German cancer registries; EGJ: esophagogastric junction; * percentages within the subgroup; LCNEC: large-cell NEC; SCNEC: small-cell NEC; NEC, nos: NEC, not otherwise specified.

**Table 2:** Anatomic localization of neuroendocrine neoplasms in comparison to adenocarcinoma or squamous cell carcinoma of the esophagus among men and women in Germany, 2009–2023.

	NEN	Subtypes of NEN			AC	SCC
Characteristic		NET	NEC	MiNEN		
Total, n	1114	109	911	94	43,492	46,661
Overlapping or unspecified anatomic localizations, n						
Overlapping	65	3	52	10	1521	2584
Unspecified	297	32	254	11	8185	11,343
Specified anatomic localizations, n	752	74	605	73	33,786	32,734
Cervical, %	2.5	1.4	2.8	1.4	0.9	6.9
Thoracic, %						
Not other specified, %	3.6	2.7	3.8	2.7	1.6	5.7
Upper, %	7.9	6.8	8.4	4.1	1.9	20.2
Middle, %	21.9	25.7	23.1	8.2	6.1	35.8
Lower, %	61.0	60.8	58.8	79.5	86.1	30.2
Abdominal, %	3.1	2.7	3.0	4.1	3.4	1.3

Legend: analyses include all German cancer registries; NEN: neuroendocrine neoplasm; NET: neuroendocrine tumor; NEC: neuroendocrine carcinoma; MiNEN: mixed neuroendocrine-non-neuroendocrine neoplasm; AC: adenocarcinoma; SCC: squamous cell carcinoma.

**Table 3:** Most recent incidence estimates of cancers (cases per million person-years) of the esophagus and esophagogastric junction by histological group in Germany, 2019–2023.

Characteristic	Men					Women				
	N	CR	SE	ASR	SE	N	CR	SE	ASR	SE
Esophagus & esophagogastric junction	47,469	231.4	1.1	148.7	1.1	14,116	67.0	0.6	36.0	0.6
**Esophagus**										
All cancers	29,455	143.6	0.8	92.3	0.8	8852	42.0	0.4	22.5	0.4
Adenocarcinomas	14,183	69.1	0.6	45.2	0.6	2883	13.7	0.3	7.4	0.3
Squamous cell carcinomas	10,875	53.0	0.5	33.9	0.5	4317	20.5	0.3	11.4	0.3
Neuroendocrine neoplasms	352	1.7	0.1	1.1	0.1	140	0.7	0.1	0.4	0.1
Other specified cancers	1361	6.6	0.2	4.1	0.2	508	2.4	0.1	1.2	0.1
Unspecified cancers	2684	13.1	0.3	8.0	0.3	1004	4.8	0.2	2.2	0.2
**Esophagogastric junction**										
All cancers	18,014	87.8	0.7	56.4	0.7	5264	25.0	0.3	13.5	0.3
Adenocarcinomas	16,071	78.4	0.6	50.5	0.6	4391	20.8	0.3	11.3	0.3
Squamous cell carcinomas	273	1.3	0.1	0.8	0.1	95	0.5	0.05	0.2	0.05
Neuroendocrine neoplasms	294	1.4	0.1	0.9	0.1	126	0.6	0.1	0.4	0.1
Other specified cancers	747	3.6	0.1	2.3	0.1	411	1.9	0.1	1.1	0.1
Unspecified cancers	629	3.1	0.1	1.9	0.1	241	1.1	0.1	0.5	0.1

Legend: analyses include all German cancer registries; CR: crude rate; SE: standard error of the rate; ASR: age-standardized rate (old European Standard population).

## Data Availability

Data used in this project can be requested from the Center for Cancer Registry Data (ZfKD) at the Robert Koch Institute, Nordufer 20, 13353 Berlin.

## Supplementary Materials

The following supporting information can be downloaded at: https://www.mdpi.com/article/10.3390/curroncol33020101/s1, Supplementary Table S1: Absolute and relative 5-year survival of patients with neuroendocrine neoplasms of the esophagus and esophagogastric junction in Germany, 2009–2023 (period approach); Supplementary Text: Adenocarcinoma and squaous cell carcinoma of the esophagus and esophagogastric junction; Supplementary Table S2: Potential determinants of missing AJCC stage among patients with neuroendocrine tumors in Germany, 2009–2023; Supplementary Table S3: Distribution of AJCC stages among 259 patients with newly diagnosed neuroendocrine tumors in Germany, 2009–2023, before and after multiple imputation; Supplementary Table S4: Effect of grading on overall survival among patients with newly diagnosed neuroendocrine tumors of the esophagus or esophagogastric junction in Germany, 2009–2023; Supplementary Table S5: Squamous cell and adenocarcinoma of the esophagus and esophagogastric junction in Germany, 2009–2023; Supplementary Table S6: Relative 5-year survival of patients with squamous cell or adenocarcinoma of the esophagus and esophagogastric junction in Germany, 2019–2023 (period approach); Supplementary Figure S1: Age distribution of male and female patients with squamous cell carcinoma, adenocarcinoma, or neuroendocrine neoplasms of the esophagus and esophagogastric junction in Germany, 2009–2013.

## Abbreviations

The following abbreviations are used in this manuscript:

AC	Adenocarcinoma
AJCC	American Joint Committee on Cancer
ASR	Age-standardized incidence rate
CR	Crude incidence rate
DCO	Death certificate only
EGJ	Esophagogastric junction
ICD-10	International Classification of Diseases and Related Health Problems, 10th edition
MANEC	Mixed adenoneuroendocrine carcinoma
MiNEN	Mixed neuroendocrine-non-neuroendocrine neoplasm
NEC	Neuroendocrine carcinoma
NEN	Neuroendocrine neoplasm
NET	Neuroendocrine tumor
RS	Relative 5-year survival
SCC	Squamous cell carcinoma
SE	Standard error
SEER	Surveillance, Epidemiology, and End Results
UICC	Union Internationale Contre le Cancer
WHO	World Health Organization
