# Supplementary material for: Neuroendocrine Neoplasms of the Esophagus and Esophagogastric Junction in Germany, 2009–2023
Source: Curr Oncol. 2026 Feb 4;33(2):101. doi: 10.3390/curroncol33020101 (PMC12939488; doi:10.3390/curroncol33020101)
Supplement: Supplementary file 1 [file curroncol-33-00101-s001.zip › curroncol-4098489-supplementary.pdf]

Inhalt

Suppl. Table S1 Absolute and relative 5-year survival of patients with neuroendocrine neoplasms of the esophagus and esophagogastric junction, Germany, 2009-2023 (period approach) 2

Supplementary text 3

Suppl. Table S2 Potential determinants of missing AJCC stage among patients with neuroendocrine tumors in Germany, 2009-2023 4

Suppl. Table S3 Distribution of AJCC stages among 259 patients with newly diagnosed neuroendocrine tumors in Germany, 2009-2023 before and after multiple imputation 5

Suppl. Table S4 Effect of grading on overall survival among patients with newly diagnosed neuroendocrine tumors of the esophagus or esophagogastric junction in Germany, 2009-2023 6

Suppl. Table S5 Squamous cell and adenocarcinoma of the esophagus and esophagogastric junction in Germany, 2009-2023 8

Suppl. Table S6 Relative 5-year survival of patients with squamous cell or adenocarcinoma of the esophagus and esophagogastric junction, Germany, 2019-2023 (period approach) 10

Suppl. Figure S1. Age distribution of male and female patients with squamous cell carcinoma, adenocarcinoma, or neuroendocrine neoplasms of the esophagus and esophagogastric junction in Germany, 2009-2013. 12

Suppl. Table S1 Absolute and relative 5-year survival of patients with neuroendocrine neoplasms of the esophagus and esophagogastric junction, Germany, 2009-2023 (period approach)

	**Absolute 5-year survival**		**Relative 5-year survival**	
**Entity**	**%**	**SE**	**%**	**SE**
NEN	20.0	1.2	22.8	1.4
NET	53.5	3.5	59.8	3.9
NET grades				
G1	75.7	4.5	83.8	5.0
G2	44.2	6.4	50.0	7.3
G3	8.0	6.8	9.0	7.6
Unknown	39.6	10.9	45.0	12.3
NET stages				
Localized	79.1	8.9	85.9	9.7
Regional	31.2	11.0	35.2	12.4
Distant	15.1	6.8	17.0	7.7
Unknown	60.2	4.3	67.9	4.9
NEC	10.9	1.1	12.5	1.3
NEC stages				
Localized	33.2	7.3	37.3	8.2
Regional	17.4	3.5	19.8	4.0
Distant	2.7	1.2	3.1	1.4
Unknown	10.6	1.6	12.2	1.8
MiNEN	21.6	3.8	25.3	4.4
MiNEN stages				
Localized	61.2	13.7	67.3	15.0
Regional	23.5	7.8	27.5	9.2
Distant	0.0		0.0	
Unknown	23.4	6.1	28.3	7.3

Legend: death-certificate-only (DCO) cases (9 out of 1329 cases were excluded leaving 1320 cases in the analysis); SE: standard error; NEN: neuroendocrine neoplasms; NET: neuroendocrine tumors; NEC: neuroendocrine carcinomas; MiNEN: mixed neuroendocrine-non-neuroendocrine neoplasms, previously termed as mixed adenoneuroendocrine carcinoma (MANEC); AJCC stages: localized, regional lymph node metaseses, distant metastases; only survival data from Schleswig-Holstein, Hamburg, Lower Saxony, Bremen, North Rhine-Westphalia, Rhineland-Palatinate, Baden-Wurttemberg, Saarland, Saxony.

Supplementary text

Imputation model

We assumed a missing at random mechanism for staging information (AJCC) and grading information of NETs and used several covariates to impute values for stage and grading. Under the assumption of missing at random, multiple imputation corrects biases that may arise in complete case analyses (Sterne et al., 2009).

The imputation used the following categorical variables that predicted missingness: AJCC stage (localized, regional lymph node metastases, distant metastases), grading (I-III), year of diagnosis, topography (unspecified, i.e. ICD-10 C15.9 or otherwise), sex, place of living (Lower Saxony or otherwise) and age at diagnosis (< 60 or ≥ 60 years) and vital status (died or survived). The imputation model further included the estimated cumulative baseline hazard as a continous variable. We approximated the cumulative baseline hazard using the Nelson-Aalen estimator (White & Royston, 2009). We ran 20 burn-in iterations before 20 multiple imputations and used the discriminant function method of classification variables. We used an noninformative prior.

Analysis model

The analysis models were Cox proportional hazards regressions for the endpoint of overall mortality including the following class variables: grading, AJCC stage, sex, and age at diagnosis (< 60 or ≥ 60 years). All variables were included as categorical variables in the model. The effect estimates of the 20 imputed data sets were pooled by Rubin's rule Rubin 1987) using using SAS PROC MIANALYZE. All multiple imputation analyses were done with SAS 9.4 (Cary, NC, USA).

References

RubinDB. Multiple imputation for nonresponse in surveys. NewYork:Wiley; 1987.

Sterne JAC. Multiple imputation for missing data in epidemiological and clinical research: potential and pitfalls. BMJ 2009;338:b2393

White IR, Royston P. Imputing missing covariate values for the Cox model. Stat Med 2009;28:1982e98.

Suppl. Table S2 Potential determinants of missing AJCC stage among patients with neuroendocrine tumors in Germany, 2009-2023

Characteristic	Patients without missing AJCC		Patients with missing AJCC	
	N	%	N	%
**Total number**	**99**		**160**	
**Sex**				
Men	59	59.6	81	50.6
Women	40	40.4	79	49.4
**Age at diagnosis (years)**				
< 50	3	3.0	16	10.0
50-59	21	21.2	26	16.3
60-69	37	37.4	51	31.9
70-79	31	31.3	49	30.6
≥ 80	7	7.1	18	11.3
**Grading**				
I	25	25.3	90	56.3
II	38	38.4	32	20.0
III	25	25.3	18	11.3
unknown	11	11.1	20	12.5
**Anatomic localization**				
Esophagus, specified	34	34.3	18	11.3
Esophagus unspecified or EGJ junction	65	65.7	142	88.8
**Federal state of living**				
Schleswig-Holstein	11	11.1	9	5.6
Hamburg	1	1.0	5	3.1
Lower Saxony	12	12.1	29	18.1
Bremen	1	1.0	1	0.6
North Rhine-Westphalia	27	27.3	65	40.6
Rhineland-Palatinate	10	10.1	7	4.4
Baden-Württemberg	21	21.2	30	18.8
Saarland	2	2.0	3	1.9
Saxony	14	14.1	11	6.9
**Year of diagnosis**				
2009-2013	11	11.1	33	20.6
2014-2018	27	27.3	46	28.8
2019-2023	61	61.6	81	50.6
**Vital status**				
Survived	48	48.5	99	61.9
Died	51	51.5	61	38.1

Legend: all analyses are based on data from Schleswig-Holstein, Hamburg, Lower Saxony, Bremen, North Rhine-Westphalia, Rhineland-Palatinate, Baden-Wurttemberg, Saarland, Saxony; percentages are column percentages;.

Suppl. Table S3 Distribution of AJCC stages among 259 patients with newly diagnosed neuroendocrine tumors in Germany, 2009-2023 before and after multiple imputation

Characteristic	Before multiple imputation		After multiple imputation	
	N	%[1)]	N[2)]	%
**AJCC stage**				
Localized	36	36.4	121.6	46.9
Regional lymphnode metast.	22	22.2	49.1	19.0
Distant metast.	41	41.4	88.4	34.1
Unknown	160			
**Grading**				
I	115	50.4	128.2	49.5
II	70	30.7	80.3	31.0
III	43	18.9	50.6	19.5
unknown	31			

Legend: all analyses are based on data from Schleswig-Holstein, Hamburg, Lower Saxony, Bremen, North Rhine-Westphalia, Rhineland-Palatinate, Baden-Wurttemberg, Saarland, Saxony; 1) percentage distribution only among cases with AJCC staging information; 2) number or patients are averaged across 20 imputations.

Suppl. Table S4 Effect of grading on overall survival among patients with newly diagnosed neuroendocrine tumors of the esophagus or esophagogastric junction in Germany, 2009-2023

Model	Cohort	Adjustment set	Hazard Ratio	95%CI
Overall mortality				
#1	Crude analysis (n=228)[1)]	Empty		
	Grade I		Ref.	
	Grade II		2.73	1.69-4.43
	Grade III		7.74	4.54-13.18
#2	Complete cases (n=88)[2)]	AJCC stage, sex, calendar period, age at diagnosis (continuous)		
	Grade I		Ref.	
	Grade II		3.16	1.22-8.19
	Grade III		3.03	1.03-8.87
#3	Multiple imputation (n=259)[3)]	AJCC stage, sex, calendar period, age group		
	Grade I		Ref.	
	Grade II		2.08	1.10-3.97
	Grade III		2.42	1.14-5.12

Legend: all analyses are based on data from Schleswig-Holstein, Hamburg, Lower Saxony, Bremen, North Rhine-Westphalia, Rhineland-Palatinate, Baden-Wurttemberg, Saarland, Saxony; 1) all patients with nonmissing information on grading; 2) all patients with nonmissing information on grading and all adjusted covariates; 3) includes the total cohort of NET patients;

Adenocarcinoma and squaous cell carcinoma of the esophagus and esophagogastric junction

Between 2009 and 2023, a total of 46,634 and 928 squamous cell carcinomas of the oesophagus and EGJ were registered in Germany. In the same period, a total of 43,492 and 54,965 adenocarcinomas of the esophagus and EGJ were registered. Adenocarcinomas of the esophagus tended to occur predominantly in the lower section of the oesophagus compared to squamous cell carcinomas of the esophagus. Squamous cell carcinomas of the EGJ were very rare compared to adenocarcinomas (Suppl. Table 5).

The most recent relative 5-year survival probabilites for patients with esophageal cancer were higher in women than in men, particularly for adenocarcinomas (squamous cell carcinomas: women +12.1 percentage points (95% CI: 7.9 to 16.3 percentage points), adenocarcinomas: women +3.7 percentage points (95% CI 1.7 to 5.7 percentage points). Even among patients with tumors of the EGJ, women with squamous cell carcinomas showed better survival than men (+6.0 percentage points (95% CI -9.8 to +21.8 percentage points), while relative survival probabilities for adenocarcinomas of the EGJ were virtually the same for men and women. The location of the squamous cell carcinoma within the esophagus had little influence on survival. There was no clear pattern regarding the anatomical localization and survival of adenocarcinomas of the esophagus. Tumors that overlapped several anatomical segments had a lower probability of survival. Higher age at diagnosis of squamous cell carcinomas and adenocarcinomas of both the esophagus and the EGJ was generally associated with a lower probability of survival (Suppl. Table 6).

Suppl. Table S5 Squamous cell and adenocarcinoma of the esophagus and esophagogastric junction in Germany, 2009-2023

Characteristic	Men		Women	
**Esophagus**				
**Squamous cell carcinoma, n**	**34265**		**12396**	
Age at diagnosis (years), n (%)				
Mean (standard deviation)	67.5	9.9	69.7	10.9
< 50	1321	3.9	420	3.4
50-59	6753	19.7	1937	15.6
60-69	11944	34.9	3889	31.4
70-79	10553	30.8	3858	31.1
80+	3694	10.8	2292	18.5
Anatomic localization (ICD-10), n (%)				
Cervical (C15.0)	1580	4.6	689	5.6
Thoracic (C15.1, C15.3-5)				
Not specified (C15.1)	1332	3.9	522	4.2
Upper portion (C15.3)	4894	14.3	1715	13.8
Mid portion (C15.4)	8317	24.3	3394	27.4
Lower portion (C15.5)	7626	22.3	2250	18.2
Abdominal (C15.2)	321	0.9	94	0.8
Overlapping (C15.8)	1935	5.7	649	5.2
Unspecified (C15.9)	8260	24.1	3083	24.9
**Adenocarcinoma, n**	36285		7207	
Age at diagnosis (years), n (%)	67.3	11.1	71.6	12.3
Mean (standard deviation)				
< 50	2173	6.0	314	4.4
50-59	7542	20.8	1040	14.4
60-69	11285	31.1	1740	24.1
70-79	10267	28.3	2062	28.6
80+	5018	13.8	2051	28.5
Anatomic localization (ICD-10), n (%)				
Cervical (C15.0)	264	0.7	51	0.7
Thoracic (C15.1, C15.3-5)				
Not specified (C15.1)	450	1.2	95	1.3
Upper portion (C15.3)	477	1.3	156	2.2
Mid portion (C15.4)	1550	4.3	513	7.1
Lower portion (C15.5)	24653	67.9	4433	61.5
Abdominal (C15.2)	977	2.7	167	2.3
Overlapping (C15.8)	1245	3.4	276	3.8
Unspecified (C15.9)	6669	18.4	1516	21.0

Suppl. Table S5 (continued)

Characteristic	Men		Women	
**Esophagogastric junction**				
**Squamous cell carcinoma, n**	**699**		**229**	
Age at diagnosis (years), n (%)				
Mean (standard deviation)	68.3	11.0	72.2	12.0
< 50	33	4.7	9	3.9
50-59	128	18.3	29	12.7
60-69	218	31.2	52	22.7
70-79	208	29.8	75	32.8
80+	112	16.0	64	28.0
**Adenocarcinoma, n**	**42809**		**12156**	
Age at diagnosis (years), n (%)				
Mean (standard deviation)	68.2	11.5	71.6	12.6
< 50	2528	5.9	675	5.6
50-59	7903	18.5	1620	13.3
60-69	12356	28.9	2642	21.7
70-79	13092	30.6	3769	31.0
80+	6930	16.2	3450	28.4

Legend: includes all German cancer registries.

Suppl. Table S6 Relative 5-year survival of patients with squamous cell or adenocarcinoma of the esophagus and esophagogastric junction, Germany, 2019-2023 (period approach)

Characteristic	Men		Women	
	Estimate	SE	Estimate	SE
**Esophagus**				
**Squamous cell carcinoma, n**	20.7	0.5	24.4	0.9
Age at diagnosis (years)				
< 50	31.8	3.5	44.2	6.0
50-59	23.6	1.3	30.8	2.4
60-69	21.6	0.9	26.9	1.6
70-79	19.7	1.0	23.5	1.5
80+	15.7	1.7	15.9	2.0
Anatomic localization (ICD-10), n (%)				
Cervical (C15.0)	19.7	2.2	25.7	3.5
Thoracic (C15.1, C15.3-5)	22.1	0.7	25.4	1.1
Not specified (C15.1)	22.3	3.0	25.0	4.8
Upper portion (C15.3)	21.5	1.4	24.3	2.3
Mid portion (C15.4)	21.6	1.1	26.6	1.7
Lower portion (C15.5)	22.9	1.2	24.4	2.0
Abdominal (C15.2)	26.6	6.5	17.0	9.2
Overlapping (C15.8)	16.7	2.1	15.0	3.0
Unspecified (C15.9)	17.5	1.1	23.2	1.8
**Adenocarcinoma**	20.1	1.8	32.2	1.2
Age at diagnosis (years)				
< 50	24.4	0.9	44.5	6.4
50-59	37.8	1.1	44.4	3.0
60-69	35.8	0.9	37.4	2.3
70-79	31.2	1.0	32.9	2.3
80+	20.6	1.5	17.4	2.2
Anatomic localization (ICD-10), n (%)				
Cervical (C15.0)	13.2	4.0	19.5	12.4
Thoracic (C15.1, C15.3-5)	25.7	3.5	34.8	1.4
Not specified (C15.1)	19.3	3.6	17.0	8.2
Upper portion (C15.3)	30.3	4.8	23.8	7.5
Mid portion (C15.4)	22.0	2.3	29.3	4.5
Lower portion (C15.5)	26.6	1.7	36.1	1.6
Abdominal (C15.2)	38.4	3.8	33.3	7.6
Overlapping (C15.8)	19.3	2.4	20.4	4.6
Unspecified (C15.9)	28.8	1.3	25.5	2.5

Suppl. Table S6 (continued)

Characteristic	Men		Women	
	Estimate	SE	Estimate	SE
**Esophagogastric junction**				
**Squamous cell carcinoma**	22.3	3.6	28.3	7.2
Age at diagnosis (years)				
< 50	47.3	18.5	.	.
50-59	37.1	10.3	35.3	19.0
60-69	21.0	6.1	38.3	13.9
70-79	22.4	7.1	29.9	13.3
80+	0.0	.	18.1	15.7
**Adenocarcinoma**	31.3	0.5	30.0	1.0
Age at diagnosis (years)				
< 50	34.6	2.1	33.5	4.1
50-59	34.5	1.1	38.6	2.5
60-69	35.9	0.9	38.2	2.0
70-79	29.6	1.0	30.9	1.8
80+	22.5	1.5	17.4	1.8

Legend: inlcudes only survival data from Schleswig-Holstein, Hamburg, Lower Saxony, Bremen, North Rhine-Westphalia, Rhineland-Palatinate, Baden-Wurttemberg, Saarland, Saxony

Suppl. Figure S1. Age distribution of male and female patients with squamous cell carcinoma, adenocarcinoma, or neuroendocrine neoplasms of the esophagus and esophagogastric junction in Germany, 2009-2013.

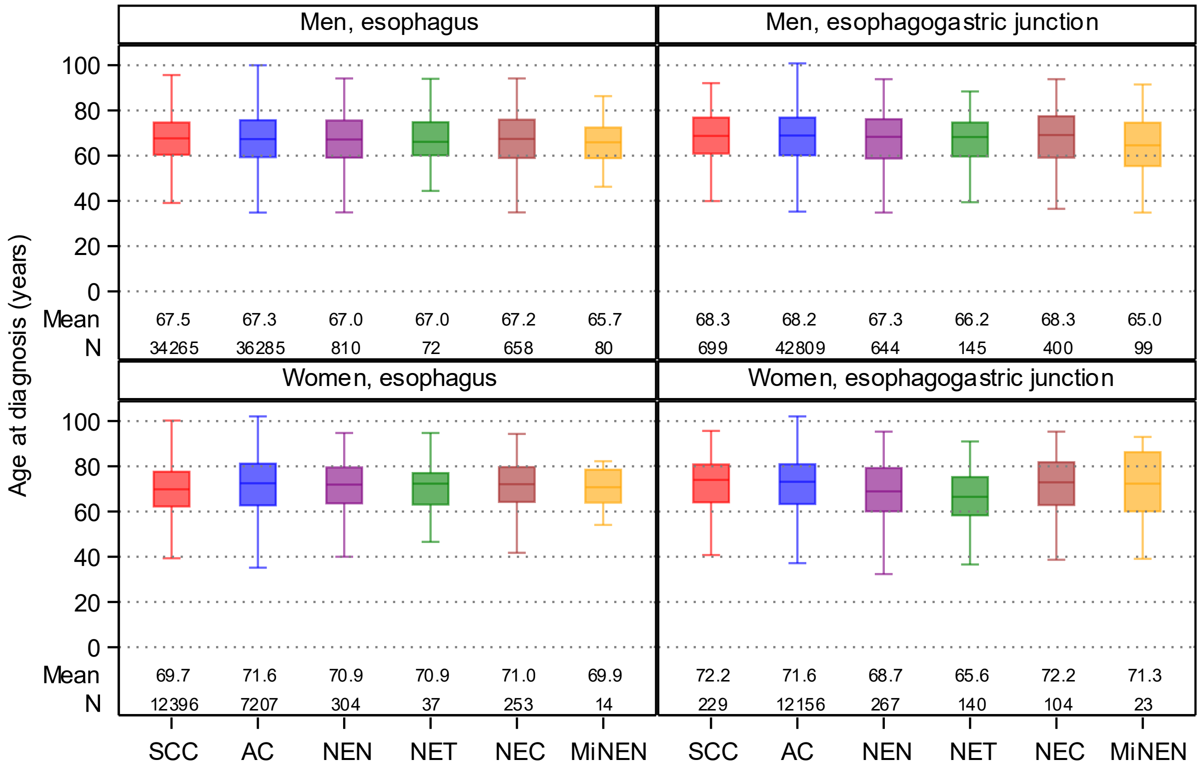

Legend: analyses include all German cancer registries; SCC: squamous cell carcinoma, AC: adenocarcinoma, NEN: neuroendocrine neoplasms overall, NET: neuroendocrine tumor, NEC: neuroendocrine carcinoma, MiNEN: mixed neuroendocrine-non-neuroendocrine neoplasms, previously termed as mixed adenoneuroendocrine carcinoma (MANEC); boxes display the interquartile ranges; lower and upper ends of the whiskers were determined by subtracting or adding 1.5 times the interquartile range from quartile 1 or to quartile 3.
